# Giant Abdominoperineal Malignant Schwannoma: An Unusual Presentation and Surgical Challenge

**DOI:** 10.1155/2015/728062

**Published:** 2015-04-02

**Authors:** Pankaj Panwar, Santosh Kumar, Shivanshu Singh, Ajjoor Shankargowda Sriharsha, Kirti Gupta

**Affiliations:** ^1^Department of Urology, PGIMER, Chandigarh 160012, India; ^2^Department of Pathology, PGIMER, Chandigarh 160012, India

## Abstract

Schwannoma is a benign tumor arising from the Schwann cells of peripheral nerves. These are usually benign but malignant transformation can occur in larger lesions. The definitive diagnosis of malignancy can only be made after final histopathological report. The literature reports large pelvic and perineal schwannomas with few being malignant. We report the first case of such giant malignant abdominoperineal schwannoma which was benign on initial biopsy but final histopathology revealed it to be malignant. In view of proximity of perineal and pelvic tumors to urogenital organs and pelvic nerves, such cases represent a challenge to surgical excision. This case brings to highlight another atypical presentation of such tumors.

## 1. Introduction

Schwannoma, also known as a neurilemmoma, arises from the peripheral nerve sheaths. These tumors occur commonly in head and neck areas [1], commonest being acoustic schwannoma. They have been reported to occur at several other places also [1–7]. These tumors usually cause symptoms due to their mass effect [5, 8]. Schwannomas occurring in presacral, perineal, and other pelvic locations come to attention only when they have increased in size to a large extent [8–10]. A search of the literature showed schwannomas occurring at above-mentioned locations have been reported uncommonly, but a schwannoma large enough to present as abdominoperineal tumor has not been reported yet. Due to their noninvasive nature and well-developed capsule, complete surgical excision is considered curative [11].

We report the case of a 61-year-old gentleman who presented to our outpatient department with complaints of painless progressive swelling in the perineal area for the past 2 years along with recent onset of constipation and LUTS (lower urinary tract symptoms). Based on initial trucut biopsy and imaging, diagnosis of giant benign abdominoperineal schwannoma probably arising from prostate was made. Following complete surgical excision, the final histopathology proved it to be a malignant peripheral nerve sheath tumor. This case highlights another atypical presentation of pelvic and perineal schwannomas and need to do complete surgical excision with preservation of important structures, as larger sized schwannomas may be malignant in nature.

## 2. Case Report

**Figure 1:**
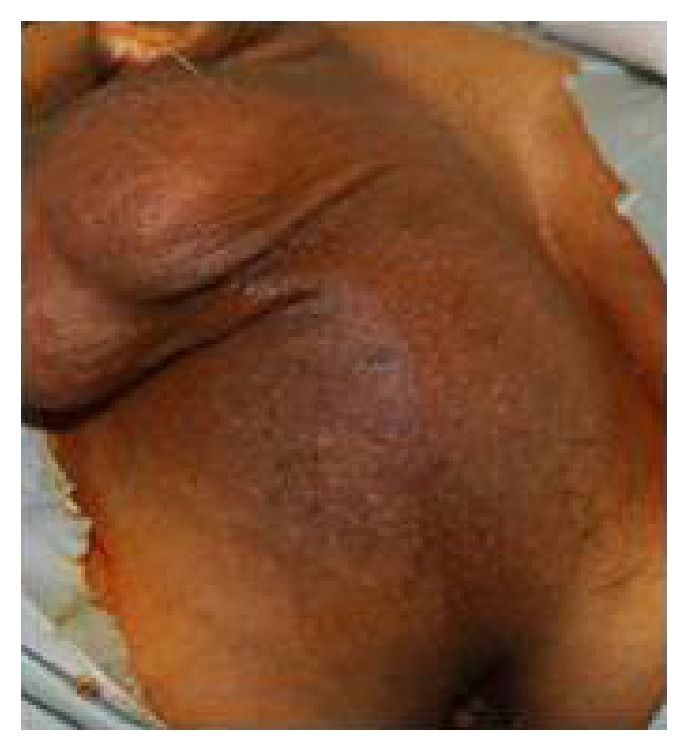
Perineal bulge produced by tumor.

**Figure 2:**
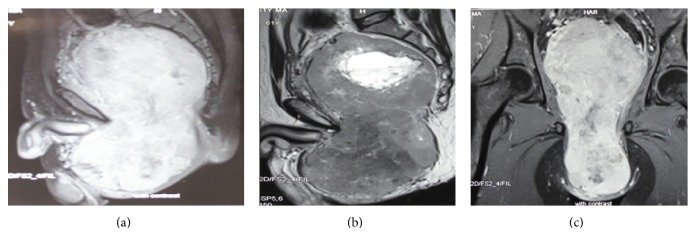
(a) T1W MRI sagittal section, (b) T2W MRI sagittal section, and (c) T1W MRI coronal section.

**Figure 3:**
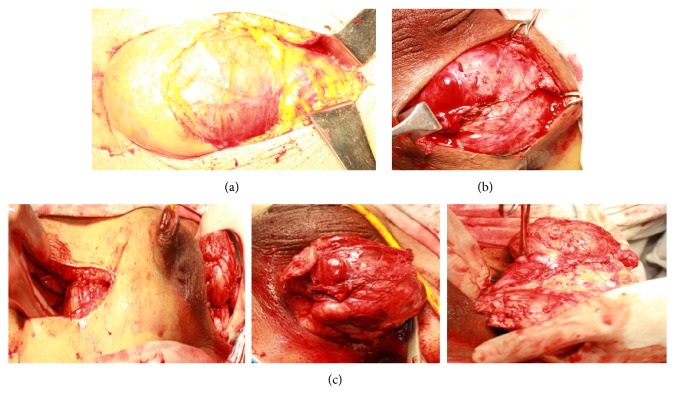
(a) Initial abdominal approach, (b) perineal approach, and (c) two approaches combined and final delivery of specimen from perineal incision.

**Figure 4:**
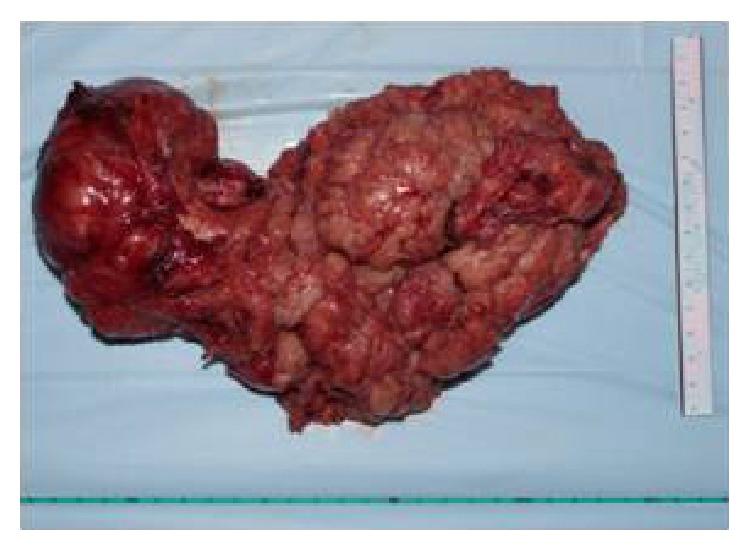
Final specimen.

**Figure 5:**
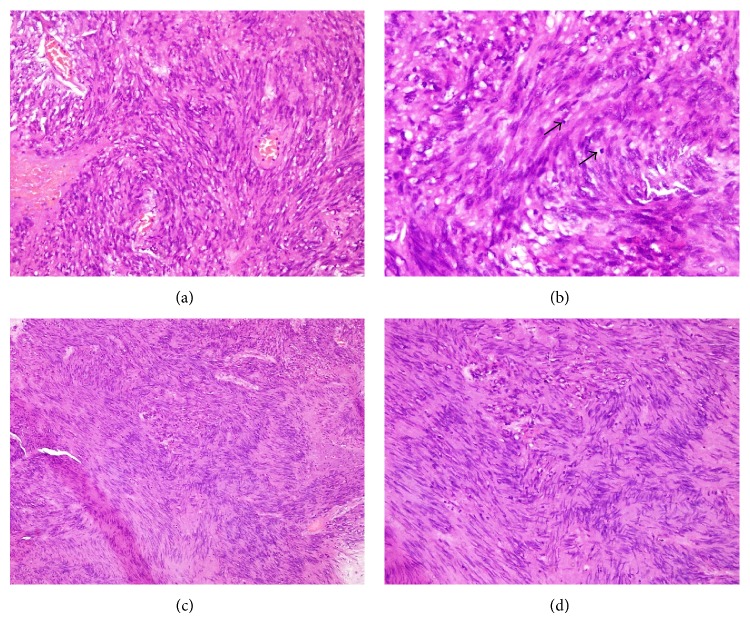
(a) Interlacing fascicles of spindle cells (H&E ×200), (b) high magnification demonstrating fusiform cells with pointed ends. Mitotic figures can also be identified (arrow), (H&E ×400), (c) & (d) focal areas featuring Vecoy body formation.

**Figure 6:**
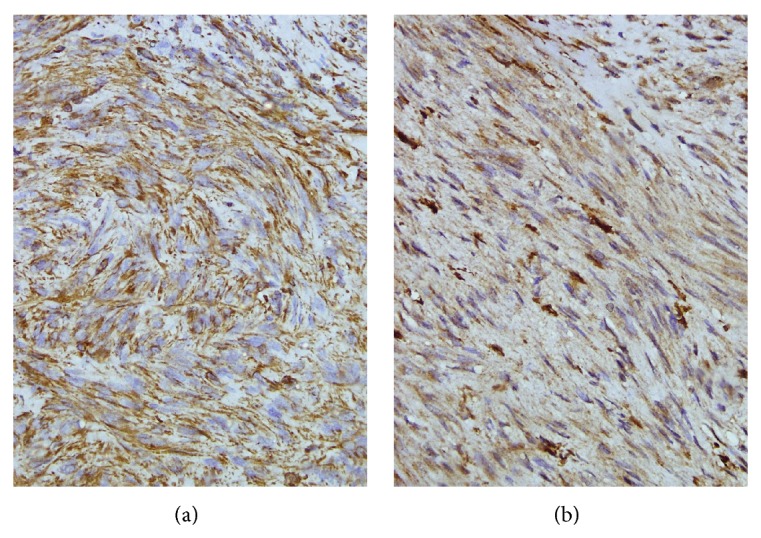
(a) Tumor cells demonstrating immunoreactivity for vimentin (antivimentin ×400), (b) tumor cells demonstrating nuclear and cytoplasmic immunoreactivity for S-100 (anti S-100 ×400).

A 61-year-old gentleman presented to our urology outpatient department with complaints of painless progressive swelling in the perineal area for the past 2 years. The swelling was around 3 cm in size to begin with and progressively increased in size to the present size of 10 cm over the last 2 years. Patient reported difficulty in sitting due to swelling. He also developed LUTS in the form of straining at urine, poor stream, intermittency, incomplete emptying, and increased daytime frequency for the past 2 months. He also complained of increasing constipation for the past 2 months. There was no history suggestive of neurofibromatosis type 1 or type 2. His general physical examination was unremarkable. Abdominal examination revealed a palpable firm swelling in the suprapubic area of size 10 × 5 cm. Examination of the perineum showed a firm immobile nontender 12 × 10 cm lump behind the scrotum with extension towards right gluteal area (Figure 1). Posteriorly the perineal lump was going up to 2 cm beyond the anal verge. On digital rectal examination (DRE), the lump had occluded majority of the rectal lumen. However, the rectal mucosa was free over the lump. Superior limit of the lump could not be felt on DRE. Prostate was not felt separately from the lump. His investigations revealed normal blood workup, with blood urea 27 mg%, serum creatinine of 0.7 mg%, and serum PSA of 0.940 ng/mL. USG pelvis and abdomen showed 12 × 10.9 cm heterogenous mass inferior to bladder with internal vascularity with anechoic areas suggestive of necrosis. Bladder was pushed anteriorly and superiorly by the mass. There were no upper tract changes. MRI abdomen showed a heterogenous solid pelvic mass on T1 image, 25 × 15 cm, which was probably arising from prostate, as prostate was not visualized separately (Figure 2(a)). Mass had central hyperintense areas on T2 image likely necrosis with increased perilesional vascularity. The mass was seen extending up to pelvic inlet superiorly and abutting the lateral pelvic wall, compressing the sigmoid colon and bladder with ill-defined planes between them (Figures 2(b) and 2(c)). The fat planes with iliac vessels were well defined. Uroflowmetry showed mild obstructive pattern. Patient underwent a TRUS guided biopsy (12 cores) from the mass, which suggested a diagnosis of benign schwannoma. Thus, with a working diagnosis of giant abdominoperineal benign schwannoma, the patient was taken up for laparotomy with tumor excision. Using meticulous dissection, it was possible to safely separate the mass from iliac vessel, sacrum, small bowels, and pelvic side walls. The pelvic plexus was also well preserved. Due to very large size of the tumor and its dumbbell shape, it could not be extracted from the abdomen itself, so a perineal incision was made and the perineal part of dissection completed from below (Figures 3(b) and 3(c)). But the tumor was densely adhered to the middle third of rectum and to bulbar urethra. In view of suspicious nature of the mass, the involved rectal wall and urethral wall were removed. The large gut continuity was restored using anal pull through and coloanal anastomosis. The urethral defect was repaired primarily. Suprapubic and perurethral catheters were placed and a diversion loop ileostomy to protect the anastomosis was also fashioned. Postoperative recovery of the patient was uneventful. The stoma started functioning from 2nd postoperative day and the patient was orally allowed. Per urethral catheter was removed on 7th postoperative day and suprapubic catheter was clamped. Patient voided well per urethrally and suprapubic catheter was also removed on postoperative day nine. Patient was discharged home on post op day ten with advice to follow up regularly. Subsequently final histopathology report was suggestive of low grade malignant peripheral nerve sheath tumor in view of presence of spindle shaped nuclei, occasional verocay bodies with mitotic counts of 2 to 3 per 10 HPF, and areas of infarction (Figures 4 and 5). IHC was positive for vimentin and S-100 (Figure 6). All margins of specimen were free of tumor. Patient is currently doing well and is under followup in our outpatient department. He is planned for ileostomy closure.

## 3. Discussion

Schwannomas are benign neoplasms arising from the peripheral nerve sheath. Men from 20 to 50 years old are most commonly affected. Schwannomas most commonly occur in the head and neck region [1] as well as flexor tendon sheaths of extremities [2]. Unusual locations such as the bladder [3], scrotum [4], and fallopian tubes have been reported [7]. Malignant transformation is usually seen in case of plexiform neurofibromatosis. Malignant degeneration of the schwannoma is rare [9, 11]. Perineal tumors are uncommon and are malignant in 66% of cases [10]. Pelvic Schwannomas account for only 1% of cases [8, 12]. They may present with nonspecific pain, palpable mass, or rectal dysfunction [8, 13]. Schwannomas account for 2% to 6% of gastrointestinal stromal tumors (GISTs), with the most common location being the stomach and the small intestine [5, 8–10]. Schwannoma of the rectum has also been reported rarely [5]. Extraintestinal Schwannomas usually are benign, are well encapsulated, and grow slowly, and thus symptoms are usually due to compression of surrounding structures. The diagnosis is delayed, and tumors may grow to a very large size before being symptomatic. Large tumors (greater than 8 to 10 cm) with cystic degeneration, calcification, interstitial fibrosis, and calcification are termed as the “ancient” variant [3, 11]. Antoni A (cellular and interlacing fascicles) and Antoni B (less cellular and myxoid), together with uniform staining for S100 protein, characterize the histological appearance of a typical schwannoma [14, 15]. Malignant schwannomas are large, are infiltrating, and are characterized histologically by perineural and intraneural spread, lesional proliferation, herniation into the lumina of the vessels and nuclear palisading [16]. In our case, the patient had vague symptoms for 2 years before being diagnosed, and the diagnosis on preoperative biopsy was suggestive of a benign schwannoma. Following resection, the final diagnosis turned out to be malignant based on the histopathological findings as described in the literature. Schwannomas are difficult to diagnose preoperatively due to a lack of distinguishing features on imaging studies such as ultrasound, CT, or MRI between benign and malignant schwannomas as well as schwannomas and other soft tissue tumors such as fibrosarcomas and liposarcomas [16]. In one study, it was noted that smooth well-defined border, ovoid, and spherical shape and location in the presacral region or the lower retroperitoneumm were the most distinguishing features of primary abdominal or pelvic schwannomas [16]. Usefulness of imaging lies in planning out therapeutic interventions. Preoperative FNAC (fine needle aspiration cytology) is of doubtful value as specimens thus obtained are frequently insufficient, and cellular pleomorphism noted in degenerative areas may be misinterpreted as malignancy [11, 12]. In our case, even a preoperative biopsy failed to diagnose the malignant behavior of the tumor. Surgery is the treatment of choice for schwannoma and is curative in benign cases. Malignant schwannomas carry a poor prognosis as they are commonly resistant to chemotherapy and radiotherapy [11]. Pelvic and perineal location with large size make surgery more risky as dissection in presacral plane can be associated with massive bleeding if the tumor capsule is adherent to the presacral venous plexus [11, 12, 14–16], although blood loss was minimal in our patient.

## 4. Conclusion

Schwannomas are usually benign tumors. Large size, unusual location, and imaging characteristics should prompt one to think of a malignant schwannoma. Perineal location is rare and abdominoperineal extent has not been reported in the available literature. Symptoms are usually due to mass effect of tumor. Preoperative diagnosis is not easy due to overlapping imaging and histopathological features with other soft tissue sarcomas. Surgery is the treatment of choice and should include excising the tumor completely and if malignant transformation is suspected, the adjacent structures invaded by the tumor may need partial or complete excision as appropriate.
